# Efficacy of Different Types of Therapy for COVID-19: A Comprehensive Review

**DOI:** 10.3390/life11080753

**Published:** 2021-07-27

**Authors:** Anna Starshinova, Anna Malkova, Ulia Zinchenko, Dmitry Kudlay, Anzhela Glushkova, Irina Dovgalyk, Piotr Yablonskiy, Yehuda Shoenfeld

**Affiliations:** 1 Almazov National Medical Research Centre, Head of the Research Department, 2 Akkuratov Str., 197341 Saint-Petersburg, Russia; 2 Medical Department, Saint Petersburg State University, 199034 Saint-Petersburg, Russia; anya.malkova.95@mail.ru (A.M.); piotr_yablonskii@mail.ru (P.Y.); yehuda.shoenfeld@sheba.health.gov.il (Y.S.); 3 St. Petersburg Research Institute of Phthisiopulmonology, 199034 Saint-Petersburg, Russia; ulia-zinchenko@yandex.ru (U.Z.); prdovgaluk@mail.ru (I.D.); 4 NRC Institute of Immunology FMBA of Russia, 115478 Moscow, Russia; D624254@gmail.com; 5 Medical Department, I.M. Sechenov First Moscow State Medical University, 119435 Moscow, Russia; 6 V.M. Bekhterev National Research Medical Center for Psychiatry and Neurology, 192019 Saint Petersburg, Russia; angela_glushkova@yahoo.com; 7 Ariel University, Kiryat HaMada 3, Ariel 40700, Israel; 8 Zabludowicz Center for Autoimmune Diseases, Sheba Medical Center, Tel-Hashomer 5265601, Israel

**Keywords:** coronavirus infection, SARS-CoV-2, COVID-19, antiviral therapy, immune therapy, cytokines, plasma, intravenous immunoglobulin IgG

## Abstract

A new coronavirus disease (COVID-19) has already affected millions of people in 213 countries. The possibilities of treatment have been reviewed in recent publications but there are many controversial results and conclusions. An analysis of the studies did not reveal a difference in mortality level between people treated with standard therapy, such as antiviral drugs and dexamethasone, and new antiviral drugs/additional immune therapy. However, most studies describe clinical improvement and a decrease in mortality among patients with severe and critical conditions, with the early initiation of additional immune therapy. Possible new targets based on viral life cycles were considered. Unfortunately, the data analysis on the efficacy of different medicine and therapy regimens among patients with COVID-19, showed little success in decreasing the mortality rate in all treatment methods. Some efficacy has been shown with an immunosuppressive therapy in small patient samples, but when a larger number of patients were analyzed the data did not differ significantly from the control groups.

## 1. Introduction

The first novel coronavirus cases were officially recorded in Wuhan, Hubei Province, China (PRC) at the end of December 2019 [1,2]. At the end of 2019, the spread of the novel coronavirus caused by the SARS-CoV-2 virus led to the death of patients in 4–22% of cases [3,4], which were associated with severe manifestations of the disease, most often in adults with concomitant pathologies [5,6,7].

There is currently no etiological treatment for coronavirus infection, and a standard therapy is based on the pathogenesis of the disease. According to the pathogenesis established by Chinese scientists, the process can be divided into three stages [8]. Coronaviruses entering the mucosa of the upper respiratory tract are likely replicated in the cells of the ciliary epithelium [9] and cause rhinitis, glossitis, and a cough with possible systemic intoxication, manifested by fever and arthralgia [10]. When overcoming the upper respiratory tract barriers, the virus enters the lungs, where it binds to the angiotensin converting enzyme (ACE) using the receptor-binding domain (RBD) S1 of the subunit of the surface S (spike) protein, which initiates virion endocytosis in the cell [11,12,13]. From the lungs, the virus enters the systemic circulation known as the viremia phase. During this stage, the virus attacks cells that also express ACE: type 2 pneumocytes in the alveolar epithelium, heart, kidney, gastrointestinal tract cells, macrophages [14,15,16], as well as the endothelium of arterial and venous vessels, smooth muscle cells in the arteries [17]. The second stage is the acute phase, characterized by organ lesions due to infection. They can be explained by several mechanisms: the direct cytotoxic effect of the virus on cells, immune-mediated complications, vascular complications, and autoimmune side effects [8,18]. The SARS-CoV-2 virus induces a weak interferon response of types I, II and III and a strong activation of the interleukin IL-1β/IL-6 pathway [19]. In the lungs, infection of type II alveolar epithelial cells activates the inflammasome, which induces the production of IL-1β [20]. IL-1β induces the secretion of IL-6 by endothelial cells and vascular smooth muscle cells, which enhance the inflammatory response [21]. In the lungs immune-competent cells infiltrate the tissue and cause an additional alteration due to excessive secretion of proteases and active forms of oxygen [15,22]. The diffuse alteration of alveoli is characterized by the desquamation of alveolar cells, formation of hyaline membranes, development of lung edema and fibrosis [17,23]. It is important to note that the acute phase, characterized by the development of pneumonia, with adequate treatment and normal functioning of the immune system is followed by a stage three recovery. In risk groups (advanced age, the presence of concomitant diseases), the immune system cannot effectively control the course of the diseases. For this reason, serious life-threatening complications such as cytokine storm and massive thrombosis may occur. In such cases, patients end up in a very serious condition and need intensive care [8].

**Figure 1:**
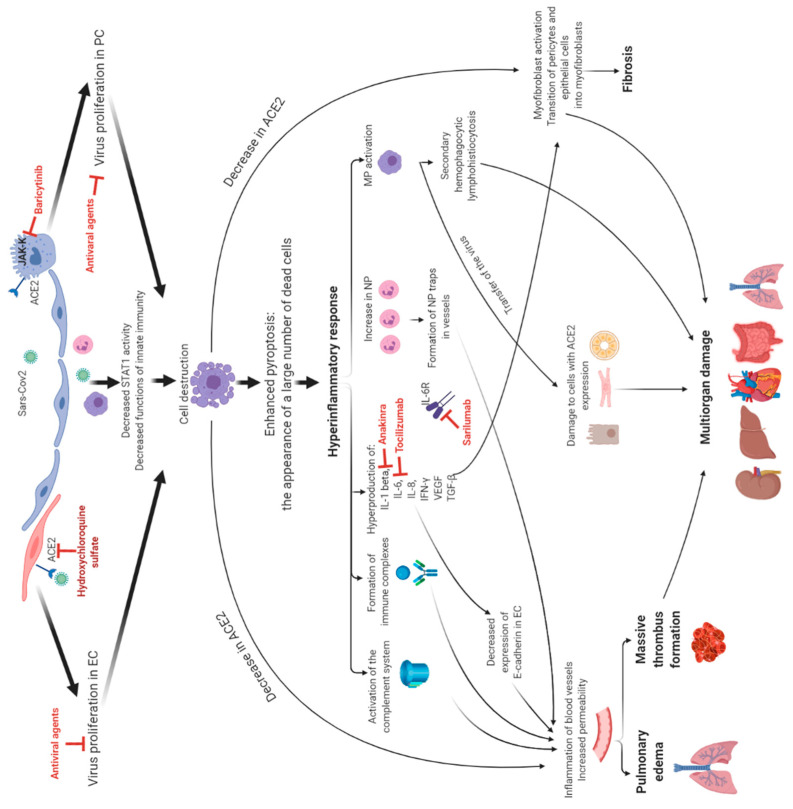
The targets of drugs used in clinical practice and their influence on pathogenic processes. Abbreviations: EC are endotheliocytes, PC are pneumocytes, NP are neutrophils, and MP are macrophages.

Therefore, existing therapy is aimed at inhibiting viral replication, as the binding to ACE2 and the activity of viral enzymes prevent the vascular and the immune complications from functioning (Figure 1). Despite the wide choice of drugs available, doubts about their efficacy, the most optimal prescription time, and patient selection criteria for certain drugs remain.

The purpose of this review is to analyze the efficacy of antiviral and immunological treatments of COVID-19.

## 2. Results and Discussion

### 2.1. Antiviral Therapies

According to the presented data (Table 1), there is only an insignificant efficacy of hydroxychloroquine sulfate when used in conjunction with azithromycin and a low efficacy as a preventive monotherapy.

A study of the efficacy of remdesivir in conjuction with COVID-19 was carried out in 53 patients with a confirmed SARS-CoV-2 virus carrier based on PCR and respiratory failure (an oxygen saturation of ≤94%/the need for oxygen support) [29]. In 68% of cases, there was an improvement in the oxygen support class, including 17 out of 30 patients who were on mechanical ventilation, and later extubated. The mortality level in the patient group who received invasive ventilation was 18% (6 out of 34) and 5% (1 out of 19) among those who did not need invasive ventilation. Furthermore, in a larger number of patients with COVID-19, another randomized trial was conducted and its findings indicated the results for the treatment of 538 patients and proved the effectiveness of the drug within 15 days of observation, compared with the control group who received a placebo (*n* = 521). However, the number of deaths in the groups did not significantly differ (7.1% versus 11.9%) [25].

One of the most significant studies with an analysis of a large number of clinical cases was devoted to the efficacy of dexamethasone in COVID-19 treatment [37]. A decrease of 10% in mortality rate was observed among patients with mechanical ventilation (29.3% vs. 41.4%). The analysis of the total mortality rate among COVID-19 patients with dexamethasone was not as significant (22.5% vs. 25.7%).

### 2.2. Immune Therapy

There are several directions that can be taken for the development of immune therapy for coronavirus infection [38]:

Monoclonal antibodies against cytokines and their receptors;

- Kinase inhibitors;
- Polyclonal antibodies by plasma therapy;
- Intravenous immunoglobulin IgG (IVIG);
- Polypeptide hormone for maturation of T cells.

#### 2.2.1. Monoclonal Antibodies against Cytokines and Their Receptors

According to the pathogenesis of hyperinflammation in COVID-19, the main participants are IL-1β and IL-6; therefore, the focus of clinical research was to study drugs which can block the signaling pathways of these molecules [19].

The results of 15 different studies using drugs to block the signaling pathways of IL-1 beta and IL-6 in patients with COVID-19 of varying levels of severity, are presented in Table 2. We can see that employing the described drugs had a beneficial effect in reducing the severity of the disease; however, in most cases, the summary indicators (survival/mortality) were similar to the control group.

It can be assumed that using cytokine inhibitors is the most appropriate way to treat patients with severe disease and hyperinflammation, where extensive organ damage is not evident and mechanical ventilation support is not needed. Other researchers have come to similar conclusions. Tocilizumab has been described as reducing fever and systemic inflammation within 5–7 days, was associated with improved oxygenation rates within 48–72 h, and also delayed the risk of intubation or mortality [54]. Analysis of clinical trials (RCT-TCZ-COVID-19, CORIMUNO-19-TOCI-1, BACC Bay Tocilizumab, and STOP-COVID-19) showed that mortality can be reduced by early medication of tocilizumab [55].

#### 2.2.2. The Kinase Inhibitors

Janus kinase inhibitors (JAK) downregulate the phosphorylation of the signal transducer and transcriptional activator (STAT) of several inflammatory proteins. Blocking the JAK inhibits the activation of the immune system and the development of inflammation (for example, the cellular response to proinflammatory cytokines such as interleukin IL-6) [56,57].

Baricitinib, a Janus kinase (JAK) inhibitor of JAK1 and JAK2 kinases, and Bruton’s tyrosine kinase (BTK), a B-cell antigen receptor signaling molecule, are currently being investigated in clinical trials. The data of the studies (*n* = 4) are shown in Table 3.

According to the analysis of these studies, the use of Janus kinase inhibitors (JAK) was associated with a clinical improvement; however, a reduction in mortality was not achieved. It should be noted that most results were obtained from small numbers of patients with differing degrees of severity which means that, for more accurate results, additional double-controlled studies with stricter inclusion criteria, a larger number of patients, and the presence of comparison groups were needed.

#### 2.2.3. Intravenous Immunoglobulin IgG (IVIG)

According to some studies, IVIG (intravenous immunoglobulin) [62,63] also achieves some efficacy in the treatment of COVID-19. Some information about clinical studies with the use of IVIG are shown in Table 4.

The obtained data are insufficient for making accurate conclusions; however, it can be noted that the use of IVIG during early stages of the disease is associated with an improvement in the clinical parameters of patients and the prognosis of the disease.

#### 2.2.4. Convalescent Plasma Transfusion

Plasma transfusion can eradicate pathogens from the circulation and neutralize ferritin and cytokines [65,66]. Convalescent plasma generated a lot of enthusiasm during early days of the COVID-19 pandemic due to its plausible mechanism of action and its easy availability from donors [67]. Information about clinical research, which was carried out by studying the efficacy of plasma convalescents with the new coronavirus infection. is provided in Table 5.

The greatest efficacy of the therapy is observed with the early use of plasma (up to 72 h) among patients with a severe level of the disease. A failure response of plasma therapy was noted in a review by Pathak et al. [67].

Perhaps the conflicting results can be explained by the lack of standards and methods for the screening of donor plasma, in search of the presence of binding and neutralizing antibodies to SARS-CoV-2, which could lead to use of plasma with a low level of antibodies [75].

#### 2.2.5. Polypeptide Hormone for Maturation of T Cells

One of the most underexplored fields of research is immunomodulation using thymosin, a polypeptide hormone used for T-cell maturation. Only two clinical trials (ChiCTR2000029541 and ChiCTR2000029806) used thymosin in combination with a standard therapy [77].

At this moment in time, it is difficult to draw conclusions on the efficacy of the immune therapy of COVID-19. However, most studies describe a clinical improvement and deacrease in mortality among patients in a severe and critical condition with the early initiation of additional immune therapy. These findings are supported by the study by Alessia Alunno et al., according to which none of the many immunomodulators had an impact on the mortality of patients; however, there is currently no final decision regarding the use of tocilizumab [78].

It is necessary to carry out a comparative analysis on the efficacy of each type of immune therapy in order to determine the effectiveness predictive factors for certain categories of patients, depending on the severity of the disease, age, concomitant diseases, and the time since the onset of symptoms.

## 3. Possible Therapy Targets for COVID Treatment

The present therapy has some advantages in its metabolic characteristics, dosages used, and potential efficacy, but “broad-spectrum” medicaments and their side effects should not be underestimated. Therefore, the research on new therapeutic targets and drugs continues to be conducted. The therapies that act on the coronavirus can be divided into several categories based on the specific pathways [79]:

- Enzymes or functional proteins for RNA synthesis and replication, for example: Nsp3 (Nsp3b, e Papain-like proteinase (PLpro)), Nsp7*Nsp8 complex, Nsp9eNsp10, Nsp14eNsp16, Nsp5 (3CLpro), Nsp12 (RdRp), Nsp13 (Helicase);
- Structural proteins for binding to human cell receptors, for example: Spike protein, E-channel, C-terminal RNA binding domain (CRBD), N-terminal RNA binding domain (NRBD);
- Virulence factors damaging the host’s innate immunity, for example: Nsp1, Nsp3c, ORF7a;
- The host’s specific receptors or enzymes, for example: TMPRSSS2, ACE2.

**Figure 2:**
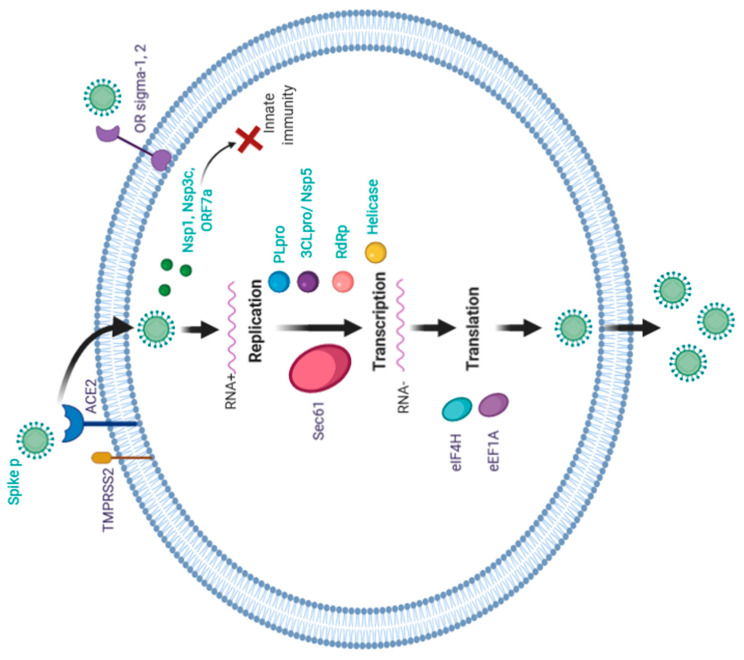
Possible therapeutic targets and their role in the life cycle of the SARS-CoV-2.

Wu C. et al. [80] conducted a virtual screening of ligands based on 21 targets (including two human targets) and selected molecules capable of inhibiting them. Gordon et al. [81] identified two classes of molecules and experimentally demonstrated their antiviral efficacy: inhibitors of protein biogenesis (zotifine, ternatin-4, and PS3061) and ligands of sigma-1 and sigma-2 receptors (haloperidol, PB28, PD-144418 and hydroxychloroquine, which is undergoing clinical trials). The authors noted the importance of the discovery of antiviral activity in sigma-1 and sigma-2 opioid receptor subtype inhibitors. It is possible that these molecules also contribute to the penetration of the virus into the cells, which may explain some neurological symptoms, in particular anosmia, because the olfactory bulb is rich in these proteins. Possible targets and their role in the life cycle of the virus are shown in the Figure 2.

According to these studies, the most promising drugs might be anti-bacterial (Chloramphenicol, Cefamandole, Tigecycline, Lymecycline, Demeclocycline, Doxycycline, Oxytetracycline, Novobiocin, Gallstonedissolving, Drug Chenodeoxycholic Acid, Cefsulodine, Rolitetracyclin, Sulfasalazine, Azlocillin, Penicillin, Pivampicillin, Hetacillin, Cefoperazone, Clindamycin, Cefmenoxime, Piperacillin, Cefpiramide, Streptomycin, Lymecycline, Tetracycline); anti-viral (Ribavirin, Saquinavir, Valganciclovir, Thymidine), anti-tumor (Idarubicin, Zotatifin, Ternatin-4, Ps3061); and anti-hypertensive (Nicardipine, Rescinnamine, Losartan, Conivaptan, Telmisartan, Iloprost, Prazosin). More detailed information is presented in Table 6.

Additionally, the scientists revealed some natural compounds (catechin compounds with an antioxidant effect and xanthones with an insecticide effect) that can block the viral life cycle in different phases. Some of these are presented in Table 7.

## 4. Conclusions

Unfortunately, the data analysis on the efficacy of different medicines and therapy regimens among patients with COVID-19 showed little success in decreasing the mortality rate through all methods. Some efficacy was shown with immunosuppressive therapy in a small number of patients, but when a larger number of patients was analyzed, the data did not differ significantly from the control groups. Furthermore, initial hopeful results concerning plasma application were ineffective in larger studies. This analysis led us to postulate that there is no evidence for the effective treatment of COVID-19 patients. Despite the presence of a great number of agents and studies conducted, no effective treatment methods were revealed.

Studies on virtual ligand screening and affinity-purification mass spectrometry revealed a wide spectrum of anti-SARS-COV2 molecules, mostly human proteins (opioid like receptors, factors of replication and translation) involved in the virus life cycle, and the molecules that inhibit them.

The most important aspect is the analysis of data on the use of various types of COVID-19 therapy in patients with a severe, critical course of the disease, especially in older age groups.

The analysis showed that, to date, there is no effective antiviral agent for the treatment of COVID-19. According to the previously obtained data, it was demonstrated that the administration of hydroxychloroquine for the treatment and prevention of coronavirus infection is not effective. On the other hand, the use of remdesevir in some patients, including those who were on invasive ventilation, showed an improvement in the course of the disease without a significant effect on mortality.

It was shown that early use of immunosuppressive agents (e.g., tocilizumab, JAK kinase inhibitors, IVIG) may have affected the severity of clinical manifestations, but did not impact mortality.

The first inspiring data on the transfusion of plasma convalescents to patients with COVID-19 were not confirmed. However, subsequent studies of this method with the formation of common standards may improve these results.

The data which demonstrated potential therapeutic targets (mainly from antiviral, antibacterial, and antihypertensive drugs, as well as human proteins involved in the virus life cycle and the molecules that inhibit them) are encouraging, but at the present moment, they are of scientific rather than practical interest.

Thus, according to the results of this analysis, none of the considered methods of COVID-19 therapy showed a significantly positive effect on mortality or a significant effectiveness in comparison with other methods, indicating the need for further research.

## Figures and Tables

**Table 1:** The results of the studies on the efficacy of antiviral drugs for COVID-19 treatment.

N	Authors and Year of Publication	The Agent Studied	Mode of Drug Administration	Number of Patients/Control Group	Observation Time, Days (Median)	Comparison of Efficiency with Control Group (%)	Conclusions
1	Cao B. et al., 2020 [24]	lopinavir/ritonavir	400/100 mg twice a day 14 days	99/control (*n* = 100)	28	Mortality 19.2 vs. 25.0	No difference
2	Li Y et al., 2020 [25]	lopinavir/ritonavir umifenovir and hydrochloride monohidrate	200/50 mg 2 times/day 7–14 days and 200 mg 3 t/day 7–14 days	34 versus 35 and control (*n* = 17)	21	Efficacy: 85.3 vs. 91.4 vs 76.5	
3	Gautret Ph. et al., 2020 [26]	Hydroxychlo-roquine sulfate	600 mg/day 10 days	20/control (*n* = 16)	14	Efficacy: 57.1 vs. 12.5	
4	Gautret P, et al., 2020 [27]	hydroxychloroquine sulfate + azithromycin	600 mg/day 10 days + 500 mg on 1-st day, further 250 mg 2nd–5th day	80/no	≥6	Efficacy: 93.0	
5	Geleris J. et al., 2020 [28]	hydroxychloroquine sulfate	600 mg on 1-st day, further 400 mg/day	811/control (*n* = 565)	22.5	Efficacy: 45.8 (no data)	
6	Grein J. et al., 2020 [29]	remdesivir	200 mg on 1-st day, further 100 mg 2nd–10th day	53/no	19	Efficacy: 47.0 (no data)	
7	WangY. et al., 2020 [30]		200 mg on 1-st day, further 100 mg 2nd–10th day	158/Placebo control (*n* = 79)	28	Efficacy: 65.0 vs. 58.0	
8	Beige JH. et al., 2020 [31]		200 mg/day for 10 days	538/placebo control 521	15	Efficacy: 62.9 vs. 52.7	
9	Goldman JD.et al, 2020 [32]		200 mg/day for 5 and 10 days	200 (5 days)/197 (10 days)	14	Efficacy: 64.0 vs. 54.0 Mortality 8.0 vs 11.0	
10	Boulware D.R. et al., 2020 [33]	hydroxychloroquine sulfate (prophylactically)	800 мг in a single dose, further 600 mg after 6 and 8 h, further 600 mg for 4 days	414 patients with asympto-matic course/407 (placebo)	14	Got sick 11.8 vs. 14.3	
11	Freedberg ED et al., 2020 [34]	famotidine	20 mg, 40 mg, 10 mg	84/control 1536	5	Mortality 10.0 vs. 22.0	
12	Horby P. at al, 2020 [35]	hydroxychloroquine sulfate	800 мг in a single dose, further 400 mg after 12 h and 6 days	1561/3155 (control)	*n*	Mortality 27.0 vs. 25.0	
13	Mather J et al., 2020 [36]	famotidine + hydroxychloroquine sulfate (*n* = 36) famotidine + azithromycin (*n* = 36) famotidine + corticosteroids (*n* = 48)	20 mg, 40 mg 7 days	83/689 (control group)	36	Mortality 21.6 vs. 39.7	

**Table 2:** The results of the studies on the efficacy of the drugs blocking the IL-1β and IL-6 signaling pathways for COVID-19 treatment.

№	Authors, Year	The Type of the Study	The Drug	The Treatment Characteristics of Patients		Conclusions
				Studied Group	Comparison Group	
1	Cavalli G et al. [39]	Retrospective cohort study	Anakinra (block IL-1 beta R)	Patients (aged ≥18 years) with COVID-19, moderate-to-severe ARDS, and hyperinflammation (*n* = 29) Standard treatment + Anankinra dose 5 mg/kg twice a day 100 mg subcutaneously 21 days	COVID-19, ARDS, and hyperinflammation Standard treatment	Decreased mortality
2	Pontali E. et al. [40]	Uncontrolled cohort study		5 patients with severe/moderate COVID-19 100 mg IV every 8 h *n* = 5	-	Faster de-escalation of the intensity of care
3	Ucciferri C et al. [41]	Retrospective cohort study	Canakinumab (block IL-1β)	300 mg subcutaneously *n* = 10	-	Faster de-escalation of the intensity of care
4	Xu X et al. [42]	Retrospective cohort study	Tocilizumab (block IL-6)	Severe or critical COVID-19 *n* = 21 4–8 mg/kg, recommended dose–400–800 mg singly 21 days	-	Faster de-escalation of the intensity of care
5	Malekzadeha R et al. [43]	Multicenter, prospective, open-label, uncontrolled		Adult patients with severe and critical COVID-19 *n* = 126 324 mg (<100 kg bodyweight) or 486 mg (≥100 kg bodyweight). 40 days	-	Faster de-escalation of the intensity of care
6	Stone JH et al. [44]	A randomized, double-blind, placebo-controlled trial		Severe acute respiratory syndrome coronavirus 2 (SARS-CoV-2) infection, hyperinflammatory states *n* = 161 4–8 mg/kg, recommended dose–400–800 mg singly 14 and 28 days	Severe acute respiratory syndrome coronavirus 2 (SARS-CoV-2) infection, hyperinflammatory states *n* = 81 Standard treatment	No difference
7	Alattar R et al. [45]	Retrospective cohort study		severe COVID-19 *n* = 25 4–8 mg/kg, recommended dose–400–800 mg singly 14 and 28 days	*n*	No difference
8	Tsai A et al. [46]	A single-center propensity-score matched cohort study		Severe COVID-19 *n* = 66 8 mg/kg, recommended dose–400–800 mg singly	Severe COVID-19 *n* = 66 Standard treatment	No difference
9	Klopfenstein T et al. [47]	a retrospective case-control study		Severe COVID-19 *n* = 20 tocilizumab (1 or 2 doses)	Severe COVID-19 *n* = 25 Standard treatment	Decreased mortality
10	Toniati P et al. [48]			severe COVID-19 *n* = 100 8 mg/kg by two consecutive intravenous infusions 12 h apart	-	Faster de-escalation of the intensity of care
11	Guaraldi G et al. [49]	Retrospective, observational cohort study		*n* = 179 intravenously at 8 mg/kg bodyweight (up to a maximum of 800 mg) in two infusions, 12 h apart, or subcutaneously at 162 mg administered in two simultaneous doses, one in each thigh (ie, 324 mg in total)	Adults (≥18 years) with severe COVID-19 *n* = 365 Standard treatment	Decreased mortality
12	Potere N. et al. [50]	Retrospective case–control study		severe COVID-19 *n* = 40 324 mg, given as two concomitant subcutaneous injections	Severe COVID-19 *n* = 40 Standard treatment (SOC)	Faster de-escalation of the intensity of care
13	Rojas-Marte G. et al. [51]	a Retrospective, case–control, Single-center study		severe to critical COVID-19 *n* = 96 4–8 mg/kg, recommended dose–400–800 mg singly 15 and 17 days	severe to critical COVID-19 *n* = 97 Standard treatment	Decreased mortality
14	Colaneri M et al. [52]	Prospective study		8 mg/kg, recommended dose–400–800 mg singly 7 days *n* = 21	*n* = 91 Standard treatment	No difference
15	Tarrytown NY. et al. [53]	Randomized Phase 2	Sarilumab (block IL-6 R)	Critical, severe COVID-19 *n* = 281 136 (200 mg)/145 (400 mg)	Critical, severe COVID-19 *n* = 77 placebo	No difference

**Table 3:** The results of the studies on efficacy of Janus kinase inhibitors for COVID-19 treatment.

№	Authors, Year	The Type of the Study	The Drug	The Treatment Characteristics of Patients		Conclusions
				Studied Group	Comparison Group	
1	Cantini F et al. [58]	Pilot study with open-label design, with no randomization and a low number of treated patients’	Baricytinib (block JAK-k)	Moderate COVID-19 4 mg/day 14 days *n* = 24	Moderate COVID-19 *n* = 24	Faster de-escalation of the intensity of care
2	Kalil AC et al. [59]	Multicenter. A randomized, double-blind ACTT-2 trial		Moderate to severe COVID-19 4 mg daily (for up to 14 days or until hospital discharge), *n* = 515	Moderate to severe COVID-19 *n* = 518 placebo	Dereased mortality
3	Cao Y et al. [60]	Small, single-blind, randomized, controlled Phase 2 trial	Ruxolitinib (block JAK-k)	Severe COVID-19 *n* = 20 5 mg orally twice daily	Severe COVID-19 *n* = 21 Placebo (vitamin C 100 mg)	No statistical difference was observed.
4	Roschewski M et al. [61]	Retrospective case series	Acalabrutinib (Bruton’s Tyrosine Kinase Inhibitors)	Severe COVID-19 *n* = 19	-	Faster de-escalation of the intensity of care

**Table 4:** The results of the studies on efficacy of IVIG for COVID-19 treatment.

№	Authors, Year	The Type of the Study	Treatment Patient Characteristics		Conclusions
			Studied Group	Comparison Group	
1	Shao Z et al. [64]	Multicenter retrospective cohort study	Critical COVID-19 *n* = 174 human Immunoglobulin (pH4) for intravenous injection 28 and 60 days	Critical COVID-19 *n* = 151	No difference
2	Zhou Z-G et al. [63]		*n* = 10 Short-term moderate-dose corticosteroid (160 mg/d) plus immunoglobulin (20 g/d)	-	Faster de-escalation of the intensity of care
3	Xie Y et al. [62]	Retrospective study	Severe or critical illness due to COVID-19 *n* = 58	-	Faster de-escalation of the intensity of care

**Table 5:** The results of the studies on efficacy of the use of convalescent plasma for COVID-19 treatment.

№	Authors, Year	The Type of Research	Treatment Patients Characteristic, *n*		Conclusions
			Studied Group	Comparison Group	
1	Simonovich VA et al. [68]	Double-blind, placebo-controlled, multicenter tria	Severe COVID-19 *n* = 228 Early administration of convalescent plasma (median titer of 1:3200 of total SARS-CoV-2 antibodies)	Severe COVID-19 *n* = 105 placebo	No difference
2	Libster R et al. [69]	A randomized, double-blind, placebo-controlled trial	Mildly ill infected older adults *n* = 80 Early administration of high-titer convalescent plasma 250 mL (IgG titer greater than 1:1000 against SARS-CoV-2 spike)	Mildly ill infected older adults *n* = 80 placebo	No statistical difference reduced the progression of COVID-19
3	Salazar E et al. [70]	Prospective, ongoing study	Severe and/or life-threatening COVID-19 *n* = 136 600 mL plasma was collected from each donor 7 and 14 days	Severe and/or life-threatening COVID-19 *n* = 251	Decreaesd mortality
4	Khamis F et al. [71]	Single-center, case series study	*n* = 11 Early therapeutic plasma exchange (TPE), 14, 28 days	Critical COVID-19 *n* = 20	Decreased mortality
5	Li L et al. [72]	Open-label, multicenter, randomized clinical trial	Severe or life-threatening COVID-19 *n* = 52 specific IgG titer ≥ 1:640; 200 mL of plasma 28 days	Severe or life-threatening COVID-19 *n* = 51	No difference
6	Gharbharan A et al. [73]	A randomized trial	*n* = 43 ≥1:80; 300 mL 15 days	*n* = 43	No statistical difference Mortality 14.0 vs. 26.0
7	Agarwal A at al [74]	Open label, parallel arm, phase II, multicentre, randomised controlled trial.	Moderate COIVD-19 *n* = 235 2 doses of 200 mL CP	*n* = 229	No statistical difference Mortality: 14.5 vs. 13.5
8	Joyner MJ et al. [75]	Open-label, Expanded Access Program (EAP) for the treatment of COVID-19 patients with human convalescent plasma.	Severe critical COVID-19 *n* = 35 150–200 mL 30 days	*n* = 322	Decreased mortality
9	Liu STH et al. [76]	Retrospective, propensity score-matched case-control study	Severe or life-threatening COVID-19 2 units of CP; 1:320 14 days *n* = 39	Severe or life-threatening COVID-19 *n* = 156	No diference

**Table 6:** Possible therapeutic targets and drugs for COVID-19 treatment.

The Group of Therapeutic Target	The Target	The Inhibiting Molecule
Blocking replication	Papain-like proteinase (PLpro)	**anti-virus drugs** (ribavirin, valganciclovir, thymidine) **anti-bacterial drugs** (chloramphenicol, cefamandole, tigecycline) **muscle relaxant drug** (chlorphenesin carbamate) **anti-tussive drug** (levodropropizine)
	3C-like main protease (3CLpro/Nsp5)	**anti-bacterial drugs** (lymecycline, demeclocycline, doxycycline, oxytetracycline) **anti-hypertensive drugs** (nicardipine, telmisartan, conivaptan)
	RNA-dependent RNA polymerase (RdRp)	**antifungal drug** itraconazole **anti-bacterial drug** novobiocin **gallstone dissolving drug** chenodeoxycholic acid **anti-allergic drug** cortisone **anti-tumor drug** idarubicin **hepatoprotective drug** silybin **muscle relaxant drug** pancuronium bromide **anticoagulant drug** dabigatran
	Helicase	**anti-bacterial drug** (lymecycline, cefsulodine, rolitetracycline) **anti-fungal drug** itraconazole **anti-HIV1 drug** saquinavir **anti-coagulant drug** dabigatran **diuretic drug** canrenoic acid
Restoring host’s innate immunity	Nsp1, Nsp3c, ORF7a	**anti-bacterial drugs** (piperacillin, cefpiramide, streptomycin, lymecycline, tetracycline)
Blocking viral structural proteins	Spike protein	**antihypertensive drugs** (rescinnamine, iloprost, prazosin) **antifungal drugs** (posaconazole, itraconazole) **anti-bacterial drug** (sulfasalazine, azlocillin, penicillin, cefsulodin) **anti-coagulant drug** dabigatran etexilate
	Interface between Spike and ACE2	Hesperidin
Blocking host‘s proteins	ACE2 protein	**antidiabetes drug** troglitazone **anti-hypertensive drug** losartan **analgesia drug** ergotamine **anti-bacterial drug** cefmenoxime **hepatoprotective drug** silybin phyllaemblicin
	TMPRSS2	**anti-bacterial drugs** (pivampicillin, hetacillin, cefoperazone, clindamycin)
	Ligands of the sigma-1,2 receptors	Haloperidol, PB28, PD-144418 and hydroxychloroquine
	Eukaryotic Translation Initiation Factor 4H (eIF4H)	Zotatifin
	Elongation factor-1A (eEF1A)	Ternatin-4
	Sec61 translocon	PS3061

**Table 7:** Some of the natural compounds considered to have an inhibiting effect on the SARS-CoV-2 life cycle.

The Plant	Scutellaria Baicalensis	Cassine Xylocarpa	Swertia Genus	Citrus Aurantium	Phyllanthus Emblica
Molecules inhibiting Sars-Cov-2	Baicalin Chrysin-7-o-b-glucuronide Wogonoside Cosmosiin	Betulonal Etexilate betulonal	Deacetylcentapicrin Triptexanthoside D 1,7-dihydroxy-3- methoxyxanthone Kouitchenside I, D	Neohesperidin Hesperidin	Phyllaemblinol Phyllaemblicin B, G7

## Data Availability

Data available in a publicly accessible repository.
